# Does back and neck pain become more common as you get older? A systematic literature review

**DOI:** 10.1186/2045-709X-20-24

**Published:** 2012-08-10

**Authors:** René Fejer, Charlotte Leboeuf-Yde

**Affiliations:** 1 The Research Department, the Spine Centre of Southern Denmark, Hospital Lillebaelt, Middelfart, Denmark; 2 Institute of Regional Health Services Research, Faculty of Health Sciences, University of Southern Denmark, Odense, Denmark

**Keywords:** Systematic literature review, Elderly population, Back pain, Low back pain, Neck pain

## Abstract

**Background:**

It is generally believed that the prevalence of back pain increases with age and as the proportion of elderly will keep rising we may be facing serious public health concerns in the future.

**Aim:**

The aim of this systematic literature review is to establish whether back pain (i.e. neck, mid-back and/or low back pain) becomes increasingly common in the older population, specifically to study 1) whether there is a significant increase in the prevalence of back pain after middle age, and 2) whether there is a significant gradually increasing prevalence of back pain with continued old age.

**Methods:**

A systematic literature search was conducted in Pubmed on articles in English, published between January 2000 and July 2011. Non-clinical studies from the developed countries with prevalence estimates on elderly people (60+) on any type of self-reported back pain and on different age groups with adequate sample sizes were included in the review. The included articles were extracted for information by two independent reviewers.

**Results:**

A total of 12 articles were included covering the entire spine. Neck pain was studied nine times, low back pain eight times, back pain three times, upper back two times and neck/shoulders once. All studies showed no significant increase of back pain with age, neither when passing from middle age (i.e. 45+ years of age) into the sixties, nor later in life. In contrast, most studies reported a decline for the oldest group.

**Conclusions:**

Back pain is no more common in the elderly population (>60 years) when compared to the middle age population. Back pain does not increase with increasing age, but seems to decline in the oldest people.

## Background

It is estimated that older people (i.e. those aged 60 and over) will account for more than 20% of the world’s population by year 2050 [1]. In addition, it is estimated that one in five of the elderly will be more than 80 years old in 2050. With the rising life expectancy a rise in the prevalence of non-communicable chronic conditions will become evident and will lead to increasing morbidity and disability [2]. According to the World Health Organization, back pain (BP) is one of the major disabling conditions among the elderly [3,4]. 

**Figure 1:**
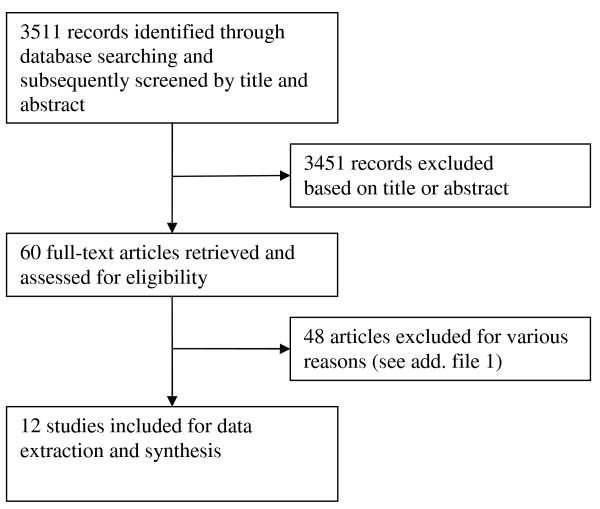
**Flow chart of search results**.

It is generally believed that BP becomes more common in old age. This appears logical, on the assumption that various types of back problems accumulate over the years. This would bring the cumulative incidence to its peak in old age. From a patho-physiological view point it also seems reasonable, as tissue degeneration increases and because the healing ability declines with age [5].

However, the literature is not clearly in favour of an increase in BP among the elderly. For example, according to a systematic critical review published in 2006 [6], non-specific BP tends to diminish in the later years whereas severe symptoms are more likely to increase. Although this is the main message of that article, it seems to relate to the development of BP over the entire lifespan. In fact, a closer look at their data on severe BP, which appear to be created on the basis of four studies, reveals no obvious increase in the prevalence of severe BP after the age of 60. Although the review failed to bring a clear answer to our concerns, their message is credible [6]. As BP is a recurring disorder for many, the true incidence would be negligible in old age, as those predestined to have BP would have experienced it already in younger years. This could mean that only very few individuals would develop first time events of BP past early adulthood or, indeed, middle age. As a consequence, the prevalence of BP would remain fairly stable over the late adult years and certainly not increase in old age.

The main aim of this literature review was therefore to investigate if BP becomes more common in old age, and specifically to study: 1) if there is a significant increase in the prevalence of BP after middle age (past 60), and 2) if there is a significant, gradually increasing prevalence of BP with continued old age.

## Methods

### Definitions

Back pain in this review includes pain anywhere in the spine, including the neck, either in a single spinal area or more widespread across the spine/neck. The older population is defined as people aged 60 and over according to the United Nation’s cut-off criterion [1]. Countries included in this review are defined as countries with an advanced economy according to the International Monetary Fund, which includes 35 countries [7].

### Search

A systematic literature search was conducted in Pubmed (http://www.pubmed.org) for a number of musculoskeletal diseases [8] published between January 1^st^ 2000 and July 1^st^ 2011. Search terms included both free text and MeSH terms and were combined by Boolean terms (AND, OR, NOT) (see Additional file 1). The following MESH terms included in this review were “neck pain”, “back pain”, and “low back pain” and were limited to include only studies containing “epidemiology”, “etiology”, or “diagnosis”. The search was restricted to English language only. No additional hand search was conducted. The retrieval of potentially relevant articles was conducted in two steps by one examiner. The first step focused on identifying relevant studies through the title and abstract. This was followed by retrieval of all full-text articles for further eligibility (see below).

### Eligibility criteria

In the present literature review, only observational studies from developed countries that reported some type of BP on people aged 60 and over were included.

The first inclusion criterion was that study samples had to represent the non-clinical population, preferably the general population, but as many elderly may live in nursing homes, such studies would also be accepted (Table 1). Also study samples obtained from general practitioners’ list of patients were accepted, as they usually cover large parts of the general population. Preferably the development of BP over age should be studied in longitudinal studies, but we suspected that only few such studies have been published, so cross-sectional studies were also accepted under the assumption that there is no cohort affect. Other criteria for inclusion were that data on BP had to be reported for specific age groups after the age of 60. If one article reported on several studies, only the latest was included and if several articles were found on the same study, then only one of them would be included. In order to make it possible for differences between estimates to reach statistical significance, only studies with a sufficiently large study sample (i.e. at least 500 people within each relevant age group) were accepted. If exact numbers were unavailable for each age group, the study was still included if the total number was deemed sufficiently large to, at least theoretically, include >500 in each group. This limit of 500 was set because it would be possible to obtain clearly non-overlapping 95% confidence intervals between two groups reporting a prevalence of 30% and 40%, respectively (upper limit 34 for 30% and lower limit of 36 for 40%).

**Table 1:** Inclusion and exclusion criteria

**Inclusion**	**Exclusion**
Original observational studies or reports; primarily cross-sectional and cohort studies	If more than one article presenting results from the same study exist then only the most pertinent article was included.
Studies reporting results specifically for various age groups on people aged 60 and over	No reviews, experimental or clinical trials, or studies with subsample of the original study sample, unless it is still a representative sample and reports new relevant information
Representative of the general population (study samples from nursing homes, etc. are accepted)	No working populations
Reported separately some type of back pain (+ divided by region)	No native/aboriginal populations
Studies from developed countries only (e.g. countries with *advanced economies* according to IMF)	No traumatic related injuries
Any type of prevalence	No secondary back pain conditions (i.e. osteoporotic fractures)
Prevalence/incidence estimates specifically on people aged 60 and over	No indirect/weighted/adjusted prevalence estimates.
In studies with results from more than one period/survey, only the most recent year was included	
At least theoretically possible that there were a minimum of 500 participants in each age group	

### Extraction of information

All core information from the included articles was extracted by an unblinded examiner and collated into two tables. The most relevant information was: Article details, method of data collection, and quality issues (sample size, response rate, method of data collection, generalisability of the study, selection bias, and how participants had been informed of the definition of the anatomical area as well as definition of pain). Further information was retrieved on relevant BP variables (BP definition, severity of BP and chronicity), relevant age ranges (age groups immediately before and after the age of 60 and subsequent age groups after 60), information with regards to the objectives: 1) If significantly higher prevalence of BP immediately after than before 60 was found (yes/no), and 2) if prevalence of BP increased significantly after the age of 60 (yes/no).

### Data analysis

Each article was reviewed independently by the two authors in order to address the two objectives. For objective 1, the cut-point of 60 years of age was sought out, but if not reported, then the two age groups closest before and after 60 were used for comparisons. Significant differences in prevalence rates for those younger and older than 60 were determined, either by observing graphs with confidence intervals, or by obtaining information in the text or tables on statistically significant differences regarding the target groups. When no such information was available but size of subgroups and prevalence proportions were reported, the 95% confidence intervals were calculated and differences considered significant if the intervals did not overlap. For objective 2, the same analysis was performed in relation to the age groups after 60 to observe if there was a statistically significant increase of prevalence estimates with increasing age.

No attempts were made at pooling prevalence estimates, not even for fairly similar BP definitions. Each study would use its own definition, and the possible increase in prevalence was considered within each study individually, thus making it unnecessary to group similar definitions or to comment on their lack of conformity. The “outcome” variable in the analysis was therefore “statistically significantly increased prevalence” yes/no, first in relation to the cut-point of 60 and thereafter for the increasing increments of age.

## Results

### Search results

In total, 3511 articles were found through the search strategy (Figure 1). Based on their titles and abstracts, 60 articles were retrieved for further reviewing. Among these potentially relevant articles, 48 were not accepted for the final reviewing process, mainly because no prevalence estimates were reported on elderly aged 60 and over or because the sample size of the age groups were too small (for a full list of excluded articles, please see Additional file 2). Hence, only twelve papers fulfilled all criteria and were thus included in this review [9-20].

### Study characteristics

Detailed information of all included studies is found in Tables 2 and 3 and briefly summarized below. In general, the method sections were well written, and all articles gave the impression of providing credible data. However, since the studies had not been designed specifically to answer our research questions, relevant outcomes were sometimes difficult to find and interpret.

**Table 2:** Study characteristics and quality issues for all included articles

**Author country year of publication**	**Sample size and response rate**	**Method of data collection**:	**Generalisability**:	**Selection bias**:	**Participants informed of the definition of BP and anatomical area**:
		**Questionnaire**	**Random sampling of general population **(**RS**) **or whole population approached **(**WP**)	**weak and confused individuals likely to have been excluded**	**In writing**
		**Interview**		**Other types of possible selection bias**	**With a drawing**
		**Physical examination**	**Response rate **>**70% **(**RRhigh**)		**Personal communication**
			**Comparison of responders and non**-**responders **(**COMP**) **or adjusted **(**ADJ**) **for non**-**responders**		
Andrianakos[9] Greece 2003	8,740	Home interview	RS	No	Personal communication
	82%		RRhigh		
			COMP		
Freburger[10]* USA 2009	2,723 (2006)	Telephone interview	RS	To some degree (telephone household interviews)	Personal communication
	57%		-		
			ADJ		
Goode[11]* USA 2010	2,809	Telephone interview	RS	To some degree (telephone household interviews)	Personal communication
	57%		-		
			COMP		
Guez[12] Sweden 2002	6,000	Telephone interview	RS	No	Not described
	72%		RRhigh		
			COMP		
Hartvigsen[13] Denmark 2004	4,486	Home interview	WP	To some degree (telephone household interviews)	Personal communication
	80.4%		-		
			-		
Keenan[14] UK 2006	16,222	Postal questionnaire	RS	No	With a drawing
	86%		RRhigh		
			COMP		
Parsons[15] UK 2007	2,504	Postal questionnaire	RS	Individuals excluded by GP if for example terminal illness, severe psychiatric disorder or severe dementia; or requested not to be involved in research	With a drawing
	60%		-		
			COMP		
Picavet[16] Netherlands 2003	3,664	Postal questionnaire	RS	No	Not described
	47%		-		
			COMP		
Santos-Eggimann[17] Switzerland 2000	3,227	Questionnaire	RS	No	With a drawing
	76% and 52% (two populations sampled)		+/- RRhigh (two populations)		
			COMP		
Strine[18] USA 2007	31,004	Household interview	RS	To some degree (household interviews)	Not described
	74%		RRhigh		
			ADJ		
Thomas[19] UK 2004	7,878	Postal questionnaire	RS	Individuals excluded by GP if for example severe psychiatric or terminal illness	With a drawing
	71%		RRhigh		
			COMP		
Webb[20] UK 2003	4,515	Postal questionnaire	RS	Unsuitable excluded by their GP	Not described
	79%		RRhigh		
			-		

*Article reporting on more than one survey in which only the latest survey was included in our review.

**Table 3:** Back pain definitions and age related information for all included articles

**Author country year of publication**	**Definition of BP **(**1**)	**Severity of BP **(**2**)	**Chronicity **(**3**)	**Age range**	**Age groups relevant for present review**	**Significantly higher prevalence of BP immediately after than immediately before 60 **(**yes**/**no**)	**Significantly higher prevalence of BP as age increases after 60 **(**yes**/**no**)	**Comments**
Andrianakos[9] Greece 2003	LBP Present or	Not described	Not described	19-	49-58; 59-68; 69-	(1) No	(1) No	Prevalence estimates decrease non-significantly in the oldest (≥69) age group for NP
	recurrent either							
	radiating or not							
	NP present or recurrent either radiating or not							
Freburger[10] USA 2009	LBP (but not NP) past yr	Not described	Chronic	21-	45-54; 55-64; 65-	(3) No	(3) No	
Goode[11] USA 2010	NP (but not LBP) past yr	Not described	Chronic	21-	45-54; 55-64; 65-	(3) No	(3) No	Prevalence estimate decreases significantly in the oldest (≥65) age group
Guez[12] Sweden 2002	NP	Not described	Chronic (continuous NP >6 months)	25-	55-64; 65-74; 75-79	(1) No for both definitions	(1) No for both definitions	Prevalence estimate for chronic NP decreases in the oldest (≥65) age groups
	NP >6 months							
						(3) No for both definitions	(3) No for both definitions	
Hartvigsen[13] Denmark 2004	BP (only) past	BP: Not described NP: stiffness or pain	Not described	70-102	70-102	N/A	(1) No	
	month							
	Neck/shoulder							
	pain (only) past month							
	Both NP and BP past month							
Keenan[14] UK 2006	NP >6 wks last 3	Any pain, swelling, and/or stiffness	Not described	55-	55-64; 65-74; ≥ 75	N/A	(1) No for both definitions	
	months							
	BP >6 wks last 3 months							
Parsons[15] UK 2007	NP past month	Moderately to severely troublesome pain	Chronic (lasting for 3 months or more)	18-101	45-54; 55-64; 65-74; 75-101	(1) No for all definitions	(1) No for all definitions	Chronic BP based on the Chronic Pain Grade scale II-IV
	upper back past					(2) No for all definitions	(2) No for all definitions	
	month					(3) No for all definitions	(3) No for all definitions	
	LBP past month							
Picavet[16] Netherlands 2003	LBP past yr	Not described in relation to age	Unclear in relation to age	25-	45-54; 55-64; 65-74; 75-	(1) No for all definitions	(1) No for all definitions	Prevalence estimates decrease non-significantly with age. For current higher BP this was significantly lower.
	Current NP							
	Current LBP							
	Current higher BP							
Santos-Eggimann[17] Switzerland 2000	LBP >7	Not described	Not described	25-74	45-54; 55-64; 65-74	(1) No for both definitions	(1) No for both definitions	Prevalence estimates increase non-significantly with age for women in both definitions, and for men with LBP >30
	cumulated days							
	past yr							
	LBP >30 cumulative days past yr							
Strine[18] USA 2007	LBP (not NP)	Not described	Not described	18-	45-54; 54-64; 65-	(1) No for all definitions	(1) No for NP and LBP only, but yes for combined NP and LBP	Prevalence estimates decrease significantly in the oldest (≥65) age group for combined NP and LBP
	≥1 day past 3							
	months							
	NP (not LBP) ≥1 day past 3 months							
	Both NP and ≥1 day past 3 months							
Thomas[19] UK 2004	NP ≥1 day past	Not described	Not described	50-	50-59; 60-69; 70-79; 80-	(1) No for both definitions	(1) No for both definitions	Prevalence estimates decreases significantly between 60-69 and 70-79 for LBP and NP
	month							
	LBP ≥1 day past							
	month							
Webb[20] UK 2003	NP ≥1wk past	Intense	Chronic	16-	45-64; 65-74; 75-	N/A	(1) An increase in NP for men, otherwise decreasing with age	Significance cannot be determined due to missing information (sample sizes). There is a general A-shape across the 3 age groups, but with a few variations.
	month							
	BP ≥1wk past month	Disabling					(2) Generally an increase from 45-64 to 65-74 in women, but not in men	
	NP and/or BP ≥1wk past month						(3) Generally an increase from 45-64 to 65-74 in women, but not in men	

* (1), (2), and (3) refer to type of pain reported.

LBP = low back pain; NP = neck pain; = back pain.

N/A = not applicable.

Nine of the twelve articles had been conducted in Europe and the remaining three in the USA. The studies used a variety of different definitions of BP, either as a single entity or a combination of pain sites and also used different recall periods, duration of pain, and intensity. Neck pain was studied nine times, low back pain eight times, BP three times, upper back twice and neck/shoulders once. Occasionally, it was not clear exactly which one of the definitions was used in relation to the age specific prevalence estimates. Age intervals ranged from 18 to 102. However, usually the upper limit was not defined. Only one study had used the World Health Organization-defined cut-point for “old”, namely 60 [19].

Five studies used either home or telephone interviews, while the rest used a questionnaire (Table 3). Overall, the generalisability was high, but some selection bias was probably likely in most of the studies, as some individuals would have been excluded for various health reasons (i.e. being weak, ill or demented). Most studies described how they defined BP for the study participants, except for four studies [12,16,18,20].

### Is there a significant increase in the prevalence of back pain after age of 60?

In all studies but one [20] was it possible to find information on or calculate statistically significance differences between the relevant age groups. It was possible to compare prevalence estimates in people just below the age 60 with those just above 60 in all but one study, which did not include study subjects younger than 70 [13]. The age intervals immediately before the age of 60 varied slightly, but, for the purpose of this review, started at 45 years of age and onwards.

As can be seen in the 7^th^ column in Table 3, none of the studies revealed a statistically significant increase of BP at this time in life and this was the case regardless the definition of BP. There was no difference in outcome between the different definitions, severity, or chronicity.

### Is there is a significant increasing prevalence of back pain with old age?

In one of the studies, significance was neither provided nor possible to calculate [20].

An increase in prevalence estimates with increasing age was reported in two studies [11,18] and possibly in a third study [20] (8th column Table 3). However, in the majority of the studies, no significant increases were found. In fact for the oldest group, estimates were generally declining in several of the studies (see last column Table 3) and significant declines were reported in three of the articles (last column Table 3). There was no difference in outcome between the different definitions, severity, or chronicity.

## Discussion

Contrary to what one may expect, self-reported BP in the general population does not get worse with old age. In fact, if anything, it seems like the prevalence of BP might decline with age. Furthermore, 60 years of age does not seem to be a critical age for BP, as there was no sign of an increased occurrence at that time of life.

### Possible explanations

These findings could be explained by a cohort effect, with people born during certain periods of time having a more or less robust physique. Only long-term (more or less life-long) longitudinal studies would be able to provide truly valid answers in relation to the course of BP, but no such studies exist. Other relevant explanations would be an increased tolerance to pain with age [21] and survival of the fittest; both resulting in lower reporting rates. A fourth explanation could be a decreased need to be physically active in old age, i.e. a reduction of pain provoking circumstances. This appears a likely explanation, as BP seems actually to diminish in the oldest old, who would be least likely to perform heavy physical activities. In three studies [10,11,18], was it obvious that the researchers had taken steps not to exclude the frail elderly (i.e. including also those with terminal illness, severe psychiatric disorders or severe dementia). It is not known if frail old people are more or less likely to have BP, so it is not known, in what direction – if any – this imbalance may push the results.

### Comparisons with other reviews

Only two previous reviews on the prevalence of BP in the elderly population have been identified [6,22]. Bressler et al. included articles between 1966 and 1999 [22]. As this current review includes articles from 2000 and onwards it would thus be interesting to compare their findings with those from the current review to see if any age related trends have changed over time. Unfortunately their objective was not to examine any trends in prevalence of low back pain with increasing age, but merely to try to establish overall prevalence estimates in the elderly population. Therefore no relevant comparisons were possible.

Age trends were, however, the main objective in Dionne et al.’s review [6]. They grouped studies by definition of BP and country of origin and dealt with any age including 65 but did not look specifically at the cut-point between middle age and old age. They reported a number of different types of curves (an increased prevalence with age, a decreased prevalence with age, a curvilinear relationship, and a flat curve) taking into account all age groups, including the younger people. In relation to middle age to old age, they found a curvilinear relationship (highest at 55) particularly for one-year period prevalence measures and for some measures of chronic pain. They also reported on the results of a weighted analysis for those aged 60 and more (with all types of BP problems); showing a relatively flat trend. A graph of a weighted calculation of severe BP predicted an increase of a few percent between the ages of 54 and 60 and about 5% between 60 and 90 years. Their study was published in 2004, and since then some additional large scale studies have been published and included in the current review. Nevertheless, our results were no different from theirs, i.e. no positive association between age and BP after middle age and into old age was found.

### Methodological considerations of the review

This review had some potential and real limitations but also some strengths. Only one electronic database (Pubmed) was included in the search and thus some relevant articles may have been missed. Based on other reviews on similar musculoskeletal conditions, who have included other electronic databases (i.e. EMBASE, CINAHL, etc.), It is therefore possible that some potentially relevant articles may have been missed [6,22]. However, unless such articles had completely different results, the occasional missing report would not affect the results, as the results were largely homogeneous.

The search strategy was also limited to the elderly population through MeSH terms. This may have lead to exclusion of some studies if for some reason they were not properly indexed in Pubmed. Finally, as only English language articles were included, any articles published in national non-English medical journals are missing in this literature review.

The review was uncritical, in the sense that although some methodological issues relating to bias were included, no attempt was made at grading the articles in relation to risk of bias. Also there were no predefined minimal criteria for acceptance in relation to quality. Had the results varied between studies, it would have been necessary to analyze study methodology and definitions, in order to understand such differences. However, this simplistic approach was apparently acceptable, as there were no difference in results across the review material, indicating that the results are fairly robust, at least in epidemiologic studies that made the effort of obtaining a relatively representative study sample.

The review was performed by two independent persons. It was always possible to reach consensus, meaning that the results probably are reproducible. Another advantage was the simplistic approach; that of dealing only with obvious difference of prevalence estimates, without confusing the picture with numerous different definitions of BP.

The heterogeneity of pain definitions is already a well known problem as it makes it difficult to compare results from different studies. It is not realistic to expect complete conformity in this area, as researchers may have had specific reasons for why they use a unique pain definition. Standardized and well defined questions are obviously important for study subjects so they know what they answer to. However, it is not important in the present review that BP definitions are all similar. Specific BP definitions would only have been necessary if there had been a need to relate prevalence estimates to the different age groups. However, as this study only attempted to find an increased prevalence over age, no attempts were made to pool results in relation to specific definitions of BP. The reason for this was that an increased prevalence of BP would be apparent regardless its definition. The decision not to concentrate on the various definitions of BP was shown to be an acceptable approach, as the findings in the present review were unequivocal across all definitions and regardless the different age-group definitions.

However, this review did not deal with the consequences of BP, nor of the duration and accompanying symptoms. Only very little information was also found on severity and chronicity. It would not be unreasonable to assume that recovery of BP is slower in old age or that some specific symptoms develop as the spine continues to degenerate. Therefore, although the prevalence of BP does not increase in old age, it is possible that specific symptoms and consequences may develop, giving a different profile of BP as the years roll on. To investigate further this concept, epidemiologic studies would have to relate age to more detailed descriptions of symptoms and consequences of BP.

## Conclusions

In relation to self-reported BP in the elderly general population, BP is not more common than in those of middle age (i.e. 45+ years of age), at least not among those who participate in surveys. Back pain does also not become increasingly common with advancing age. Thus, although BP is a common and troublesome condition in the general population, it does not seem to become increasingly common with old age, which from a public health perspective is good news.

## Competing interests

The authors declare that they have no competing interests.

## Authors’ contributions

RF and CLY planned the design of the study. RF conducted the literature search. Both authors designed the checklists, reviewed the literature and interpreted the data. RF wrote the initial draft of the manuscript. Both authors participated in the manuscript preparation. Both authors read and approved the final manuscripts.

## Supplementary Material

Additional file 1 **Search strategy. **Search strategy used in Pubmed (http://www.pubmed.org). Click here for file

Additional file 2 **Overview of excluded articles. **All retrieved articles that were initially considered of relevance based on their title and/or abstract, but subsequently excluded because inclusion criteria were not met. Click here for file
