# Supplementary material for: Does back and neck pain become more common as you get older? A systematic literature review
Source: Chiropr Man Therap. 2012 Aug 10;20:24. doi: 10.1186/2045-709X-20-24 (PMC3526387; doi:10.1186/2045-709X-20-24)
Supplement: Additional file 1 — Search strategy. Search strategy used in Pubmed (http://www.pubmed.org). [file 2045-709X-20-24-S1.doc]

Additional file

**Additional file 1. Search strategy, Pubmed (www.pubmed.org).**

(("Musculoskeletal Diseases/epidemiology"[Mesh] OR “musculoskeletal”[All Fields]) OR ("Neck Pain/epidemiology"[Mesh] OR "Back Pain/epidemiology"[Mesh] OR "Low Back Pain/epidemiology"[Mesh]))

AND

("Prevalence"[Mesh] OR "Incidence"[Mesh] OR "Cross-Sectional Studies"[Mesh] OR "Longitudinal Studies/statistics and numerical data"[Mesh])

AND

("humans"[MeSH] AND (Review[ptyp] OR Government Publications[ptyp] OR Journal Article[ptyp] OR Technical Report[ptyp])

AND

English[lang]

AND

("aged"[MeSH] OR "aged, 80 and over"[MeSH] OR “elderly”[All Fields])

AND

("2000/01/01"[PDAT] : "2011/07/01"[PDAT]))
