# Supplementary material for: Does back and neck pain become more common as you get older? A systematic literature review
Source: Chiropr Man Therap. 2012 Aug 10;20:24. doi: 10.1186/2045-709X-20-24 (PMC3526387; doi:10.1186/2045-709X-20-24)
Supplement: Additional file 2 — Overview of excluded articles. All retrieved articles that were initially considered of relevance based on their title and/or abstract, but subsequently excluded because inclusion criteria were not met. [file 2045-709X-20-24-S2.doc]

| **Additional file 2.** Excluded articles. | | |
| --- | --- | --- |
| **Reference** |  | **Main reason for exclusion** |
| Baek SR, Lim JY, Lim JY, Park JH, Lee JJ, Lee SB, Kim KW, Paik NJ: Prevalence of musculoskeletal pain in an elderly Korean population: results from the Korean Longitudinal Study on Health and Aging (KLoSHA). Arch Gerontol Geriatr 2010, 51:e46-e51 |  | Sizes of ages groups too small |
| Bergman S, Herrström P, Högström K, Petersson IF, Svensson B, Jacobsson LT. Chronic musculoskeletal pain, prevalence rates, and sociodemographic associations in a Swedish population study. J Rheumatol. 2001 Jun;28(6):1369-77 |  | No specific prevalence estimates on elderly aged 60 and over. |
| Bingefors K, Isacson D. Epidemiology, co-morbidity, and impact on health-related quality of life of self-reported headache and musculoskeletal pain--a gender perspective. Eur J Pain. 2004 Oct;8(5):435-50. |  | No specific prevalence estimates on elderly aged 60 and over. |
| Björck-van Dijken C, Fjellman-Wiklund A, Hildingsson C. Low back pain, lifestyle factors and physical activity: a population based-study. J Rehabil Med. 2008 Nov;40(10):864-9. |  | No actual population-based prevalence estimates provided on elderly aged 60 and over. |
| Blyth FM, March LM, Cousins MJ. Chronic pain-related disability and use of analgesia and health services in a Sydney community. Med J Aust. 2003 Jul 21;179(2):84-7 |  | No specific prevalence estimates on elderly aged 60 and over. |
| Bot SD, van der Waal JM, Terwee CB, van der Windt DA, Schellevis FG, Bouter LM, Dekker J. Incidence and prevalence of complaints of the neck and upper extremity in general practice. Ann Rheum Dis. 2005 Jan;64(1):118-23 |  | No specific prevalence estimates on elderly aged 60 and over. |
| Carmona L, Ballina J, Gabriel R, Laffon A: The burden of musculoskeletal diseases in the general population of Spain: results from a national survey. Ann Rheum Dis 2001, 60:1040-1045 |  | Sizes of ages groups too small |
| Cecchi F, Debolini P, Lova RM, Macchi C, Bandinelli S, Bartali B, Lauretani F, Benvenuti E, Hicks G, Ferrucci L: Epidemiology of back pain in a representative cohort of Italian persons 65 years of age and older: the InCHIANTI study. Spine (Phila Pa 1976) 2006, 31:1149-1155. |  | No age group below the age of 60 |
| Chiu TT, Leung AS: Neck pain in Hong Kong: a telephone survey on prevalence, consequences, and risk groups. Spine (Phila Pa 1976) 2006, 31:E540-E544 |  | No comparable age groups |
| Croft PR, Lewis M, Papageorgiou AC, Thomas E, Jayson MI, Macfarlane GJ, Silman AJ. Risk factors for neck pain: a longitudinal study in the general population. Pain. 2001 Sep;93(3):317-25. |  | No specific prevalence estimates on elderly aged 60 and over. |
| Damborg F, Engell V, Andersen M, Kyvik KO, Thomsen K. Prevalence, concordance, and heritability of Scheuermann kyphosis based on a study of twins. J Bone Joint Surg Am. 2006 Oct;88(10):2133-6. |  | No specific prevalence estimates on elderly aged 60 and over. |
| D'Astolfo CJ, Humphreys BK. A record review of reported musculoskeletal pain in an Ontario long term care facility. BMC Geriatr. 2006 Mar 23;6:5. |  | No specific prevalence estimates on elderly aged 60 and over. |
| Denard PJ, Holton KF, Miller J, Fink HA, Kado DM, Marshall LM, Yoo JU: Back pain, neurogenic symptoms, and physical function in relation to spondylolisthesis among elderly men. Spine J 2010, 10:865-873 |  | No comparable age groups |
| Di Iorio A, Abate M, Guralnik JM, Bandinelli S, Cecchi F, Cherubini A, Corsonello A, Foschini N, Guglielmi M, Lauretani F, Volpato S, Abate G, Ferrucci L. From chronic low back pain to disability, a multifactorial mediated pathway: the InCHIANTI study. Spine (Phila Pa 1976). 2007 Dec 15;32(26):E809-15 |  | No age group below the age of 60 |
| Docking RE, Fleming J, Brayne C, Zhao J, Macfarlane GJ, Jones GT: Epidemiology of back pain in older adults: prevalence and risk factors for back pain onset. Rheumatology (Oxford) 2011, 50:1645-1653 |  | Sizes of ages groups too small |

| **Additional file 2 (cont’d).** Excluded articles. | | |
| --- | --- | --- |
| **Reference** |  | **Main reason for exclusion** |
| Goubert L, Crombez G, De B, I: Low back pain, disability and back pain myths in a community sample: prevalence and interrelationships. Eur J Pain 2004, 8:385-394 |  | Sizes of ages groups too small |
| Gross DP, Ferrari R, Russell AS, Battié MC, Schopflocher D, Hu RW, Waddell G, Buchbinder R. A population-based survey of back pain beliefs in Canada. Spine (Phila Pa 1976). 2006 Aug 15;31(18):2142-5 |  | No specific prevalence estimates on elderly aged 60 and over. |
| Gummesson C, Isacsson SO, Isacsson AH, Andersson HI, Ektor-Andersen J, Ostergren PO, Hanson B; Malmö Shoulder-Neck Study group. The transition of reported pain in different body regions--a one-year follow-up study. BMC Musculoskelet Disord. 2006 Feb 23;7:17 |  | No specific prevalence estimates on elderly aged 60 and over. |
| Hartvigsen J, Christensen K, Frederiksen H. Back pain remains a common symptom in old age. a population-based study of 4486 Danish twins aged 70-102. Eur Spine J. 2003 Oct;12(5):528-34. Epub 2003 May 14. |  | Results included in another article (PMID 15129076) |
| Hartvigsen J, Christensen K: Pain in the back and neck are with us until the end: a nationwide interview-based survey of Danish 100-year-olds. Spine (Phila Pa 1976) 2008, 33:909-913 |  | Sizes of ages groups too small |
| Hartvigsen J, Frederiksen H, Christensen K: Back and neck pain in seniors-prevalence and impact. Eur Spine J 2006, 15:802-806. |  | No comparable age groups |
| Hicks GE, Gaines JM, Shardell M, Simonsick EM: Associations of back and leg pain with health status and functional capacity of older adults: findings from the retirement community back pain study. Arthritis Rheum 2008, 59:1306-1313. |  | Sizes of ages groups too small |
| Hill J, Lewis M, Papageorgiou AC, Dziedzic K, Croft P. Predicting persistent neck pain: a 1-year follow-up of a population cohort. Spine (Phila Pa 1976). 2004 Aug 1;29(15):1648-54 |  | No specific prevalence estimates on elderly aged 60 and over. |
| Huisstede BM, Wijnhoven HA, Bierma-Zeinstra SM, Koes BW, Verhaar JA, Picavet S. Prevalence and characteristics of complaints of the arm, neck, and/or shoulder (CANS) in the open population. Clin J Pain. 2008 Mar-Apr;24(3):253-9. |  | No specific MSK condition reported on elderly aged 60 and over. |
| Hüppe A, Müller K, Raspe H. Is the occurrence of back pain in Germany decreasing? Two regional postal surveys a decade apart. Eur J Public Health. 2007 Jun;17(3):318-22. Epub 2006 Sep 23 |  | No specific prevalence estimates on elderly aged 60 and over. |
| Ihlebaek C, Eriksen HR, Ursin H. Prevalence of subjective health complaints (SHC) in Norway. Scand J Public Health. 2002;30(1):20-9. |  | No specific prevalence estimates on elderly aged 60 and over. |
| Jacobs JM, Hammerman-Rozenberg R, Cohen A, Stessman J: Chronic back pain among the elderly: prevalence, associations, and predictors. Spine (Phila Pa 1976) 2006, 31:E203-E207 |  | Sizes of ages groups too small |
| Johannes CB, Le TK, Zhou X, Johnston JA, Dworkin RH. The prevalence of chronic pain in United States adults: results of an Internet-based survey. J Pain. 2010 Nov;11(11):1230-9. |  | No specific prevalence estimates on elderly aged 60 and over. |
| Kalichman L, Li L, Kim DH, Guermazi A, Berkin V, O'Donnell CJ, Hoffmann U, Cole R, Hunter DJ. Facet joint osteoarthritis and low back pain in the community-based population. Spine (Phila Pa 1976). 2008 Nov 1;33(23):2560-5. |  | No specific MSK condition reported on elderly aged 60 and over. |
| Knox SA, Harrison CM, Britt HC, Henderson JV. Estimating prevalence of common chronic morbidities in Australia. Med J Aust. 2008 Jul 21;189(2):66-70. |  | No specific prevalence estimates on elderly aged 60 and over. |
| Makris UE, Fraenkel L, Han L, Leo-Summers L, Gill TM. Epidemiology of restricting back pain in community-living older persons. J Am Geriatr Soc. 2011 Apr;59(4):610-4 |  | No specific prevalence estimates on elderly aged 60 and over. |
| Meyer T, Cooper J, Raspe H: Disabling low back pain and depressive symptoms in the community-dwelling elderly: a prospective study. Spine (Phila Pa 1976) 2007, 32:2380-2386 |  | No comparable age groups |

| **Additional file 2 (cont’d).** Excluded articles. | | |
| --- | --- | --- |
| **Reference** |  | **Main reason for exclusion** |
| Miro J, Paredes S, Rull M, Queral R, Miralles R, Nieto R, Huguet A, Baos J: Pain in older adults: a prevalence study in the Mediterranean region of Catalonia. Eur J Pain 2007, 11:83-92 |  | Sizes of ages groups too small |
| Natvig B, Bruusgaard D, Eriksen W. Localized low back pain and low back pain as part of widespread musculoskeletal pain: two different disorders? A cross-sectional population study. J Rehabil Med. 2001 Jan;33(1):21-5. |  | No specific prevalence estimates on elderly aged 60 and over. |
| Natvig B, Ihlebaek C, Grotle M, Brage S, Bruusgaard D: Neck pain is often a part of widespread pain and is associated with reduced functioning. Spine (Phila Pa 1976) 2010, 35:E1285-E1289 |  | Sizes of ages groups too small |
| Puts MT, Deeg DJ, Hoeymans N, Nusselder WJ, Schellevis FG. Changes in the prevalence of chronic disease and the association with disability in the older Dutch population between 1987 and 2001. Age Ageing. 2008 Mar;37(2):187-93. Epub 2008 Feb 4. |  | No specific prevalence estimates on elderly aged 60 and over. |
| Raspe H, Matthis C, Croft P, O'Neill T; European Vertebral Osteoporosis Study Group. Variation in back pain between countries: the example of Britain and Germany. Spine (Phila Pa 1976). 2004 May 1;29(9):1017-21; |  | No specific prevalence estimates on elderly aged 60 and over. |
| Ritzwoller DP, Crounse L, Shetterly S, Rublee D. The association of comorbidities, utilization and costs for patients identified with low back pain. BMC Musculoskelet Disord. 2006 Sep 18;7:72. |  | No actual population-based prevalence estimates provided on elderly aged 60 and over. |
| Rustøen T, Wahl AK, Hanestad BR, Lerdal A, Paul S, Miaskowski C. Age and the experience of chronic pain: differences in health and quality of life among younger, middle-aged, and older adults. Clin J Pain. 2005 Nov-Dec;21(6):513-23. |  | No actual population-based prevalence estimates provided on elderly aged 60 and over. |
| Salaffi F, De AR, Grassi W: Prevalence of musculoskeletal conditions in an Italian population sample: results of a regional community-based study. I. The MAPPING study. Clin Exp Rheumatol 2005, 23:819-828. |  | Sizes of ages groups too small |
| Stranjalis G, Tsamandouraki K, Sakas DE, Alamanos Y: Low back pain in a representative sample of Greek population: analysis according to personal and socioeconomic characteristics. Spine (Phila Pa 1976) 2004, 29:1355-1360 |  | Sizes of ages groups too small |
| Suka M, Yoshida K: The national burden of musculoskeletal pain in Japan: Projections to the year 2055. Clin J Pain 2009, 25:313-319 |  | Not a representative sample of the target population. |
| Tsuji T, Matsuyama Y, Sato K, Hasegawa Y, Yimin Y, Iwata H. Epidemiology of low back pain in the elderly: correlation with lumbar lordosis. J Orthop Sci. 2001;6(4):307-11 |  | Not a representative sample of the target population. |
| Vogt MT, Simonsick EM, Harris TB, Nevitt MC, Kang JD, Rubin SM, Kritchevsky SB, Newman AB: Neck and shoulder pain in 70- to 79-year-old men and women: findings from the Health, Aging and Body Composition Study. Spine J 2003, 3:435-441 |  | No comparable age groups |
| Walker BF, Muller R, Grant WD. Low back pain in Australian adults: prevalence and associated disability. J Manipulative Physiol Ther. 2004 May;27(4):238-44 |  | Sizes of ages groups too small |
| Wijnhoven HA, de Vet HC, Picavet HS. Explaining sex differences in chronic musculoskeletal pain in a general population. Pain. 2006 Sep;124(1-2):158-66. Epub 2006 May 22. |  | No specific prevalence estimates on elderly aged 60 and over. |
| Wijnhoven HA, de Vet HC, Picavet HS. Prevalence of musculoskeletal disorders is systematically higher in women than in men. Clin J Pain. 2006 Oct;22(8):717-24 |  | No specific prevalence estimates on elderly aged 60 and over. |
| Yaron M, Caspi D, Kaufman I, Zemach M, Grotto I, Altman R: Estimation of the prevalence of rheumatic diseases in Israel. Semin Arthritis Rheum 2011, 40:473-478 |  | Sizes of ages groups too small |
